# Diseases of the Eye

**Published:** 1837

**Authors:** 


### Art. V.—Diseases of the Eye.

*A Manual of Diseases of the Eye.* By S. Littell, Jr., M. D., one of the Surgeons of the Will's Hospital for the Blind and Lame, &c. &c. Phila. 1837. pp. 255.

This is a neat little volume, which we beg leave to recommend, as a comprehensive and useful hand book. Many works, especially the European, on maladies of the eye, are bulky, and from the number of their plates expensive. Dr. Littell's Manual is not liable to these objections; and at the same time contains a great deal of useful matter. All who have been conversant with the larger works on ophthalmic surgery, must have noticed with what exceeding prolixity many of them are written. Mr. Guthrie, for example, one of the ablest ophthalmic surgeons of the age, in his published lectures, has devoted 125 large octavo pages, to the subject of Artificial Pupil; and no less than 226 to Cataract. Such a tracing down of abandoned operations and obsolete theories, may be well enough in a historical treatise, and should be studied by those who design to make themselves occulists *par excellence*, but will never be read with interest or profit by the great mass of our physicians. Indeed, in reference to them, such books are rather injurious than beneficial; as they present the subject of diseases of the eye in such a discouraging aspect, that numbers are actually deterred from their study. The digest before us, is liable to none of these objections, while it is sufficiently full on the diagnosis, pathology and treatment of most of the maladies of that important organ, to enable any one acquainted with its anatomy and physiology, to treat most of them with success.

Guided by the lights of general anatomy, Dr. L. has aimed at classing diseases of the eye, according to their seats, in the different tissues. It must be admitted, however, that inflammation and most organic lesions, are seldom confined to one tissue, although they may have had such an origin.

Our author commences with inflammation of the orbit, seated in the cellular substance, the periosteum, or even the bones. The eye-ball is rendered prominent, there is sense of fullness, head-ache, and frequently delirium. An active, antiphlogistic treatment is required; but suppuration often occurs, and after the evacuation of the pus, the patient remains blind, from the inflammation having extended to the retina or optic nerve. We believe this affection to be rare, as we have met with no well marked case, except from mechanical injury.

The orbit is the seat of a variety of tumours, generally of the encysted kind. They cause more or less protrusion of the globe of the eye, and generally change the axis of vision. When the latter affection does not exist, we may infer that the tumour occupies the bottom of the socket on all sides. We have met with one case of this kind. The eye-ball was so much pushed out as to permit the eye-lids to close behind it. The tumour had grown round the optic nerve, which, of course, was greatly stretched by the projection forward of the globe. The eye was blind, but the pupil contracted, from the exposure of the other to a strong light, and became widely expanded, when that eye was in the dark. Variations of light had no effect on the diseased eye, showing, that it is not by the action of light upon the iris, but the retina, that the dimensions of the pupil are made to vary. Much dissection was necessary to the removal of this tumour, and so intense an inflammation followed, as to show, that extirpation of the globe should have been preferred to the excision of the tumour from behind it.

Wounds of the eye and its appendages come next in order. Blows, from the great amount of loose, cellular substance, are apt to be followed by extensive ecchymosis. In former times, this injury was very common in the west, from the great prevalence of a practice, which has become much less prevalent with an abatement of the heroic spirit. A favorite remedy, in those days, was the application of a slice of raw beef, or half a chicken cut in two alive! It is remarkable, how little inflammation of the eye-ball frequently follows on blows of this kind. The great extravasation seems to prevent it. But blows often produce a more serious effect—that is, immediate blindness, and this may arise, as we have seen, from three causes: first, an effusion of blood into the chambers of the eye, giving the appearance of a widely expanded pupil; a case of which, lately fell under our observation, in a seamstress, from a blow by a button; the blindness was perfect, but in 8 or 10 days the effused blood was absorbed, and the irritative amaurosis ceased;—second, the concussion sometimes destroys the sensibility of the optic nerve instantly, but such a gutta serena should not be regarded as hopeless;—third, we have more than once seen cataract form in a single day, after a blow over the orbit.

We have lately met with two cases of lacerated wounds of the sclerotica. The vitreous humour did not escape, and the inflammation was not very great, and still both produced amaurosis. In one the loss of visual sensibility was instantaneous. Such an effect could only be produced by the concussion imparted to the retina. On the cure of burns of the eye, our author says:—

"Burns, arising from whatever cause, should be treated in the first instance by some tepid, bland collyrium, which after a few days, may be exchanged for the solution just mentioned (*nitr. argenti;*) if the injury be extensive, general and local depletion, saline laxatives, &c. will also be indicated, and the utmost care will be required to guard against inflammation of the more deeply seated tissues. When the heat has been so intense as to destroy the texture of the conjunctiva, granulations arise from the inflamed cellular membrane, and adhesions are apt to form between the palpebra and the globe. To prevent such an occurrence, it will be proper to separate any recently formed bands, and to apply the nitrate of silver in substance to the inner surface of the eyelids."—p. 8.

In his remarks on wounds of the cornea, our author speaks chiefly, of such as penetrate that tunic, but those which do not pass through the membrane are far more common. In cities like Cincinnati, which manufacture stone, iron, brass, and other metals, such injuries are extremely common, and very often produce blindness, by leaving an opake eschar in front of the pupil. The fragment which is thrown off under the chissel, or by the turning lathe, is often very hot when it strikes the eye, from the pressure which detaches and projects it, so that a burn is superadded to a lacerated wound. As such fragments are angular, they are frequently imbedded in the laminæ of the cornea, in a way that renders it difficult to remove them; and too often the patient makes no application for relief until considerable inflammation has arisen. For the removal of these spiculæ, the inexperienced operator will frequently use nothing but a probe, with which he will very often fail. On this subject, the instructions of Mr. Guthrie, adopted by our author, are excellent. A curved, or scarpa's cataract needle, should be chosen, and with this the foreign body should be literally "dug out." If, in doing this, the cornea should be punctured, and a portion of the aqueous humor escape, no serious consequences will ensue. In the performance of this little operation, a strong light is necessary, and the eye-ball should be held as still as in the operation for cataract. We shall only add, that, occasionally, fragments of iron, by their black oxide, will stain the cornea, and that the operator, if unaware of this fact, may continue his efforts, when they can no longer be of any avail. If it be thought, that we have dwelt too long on the treatment of this accident, let it be recollected, that the fate of the patient's sight, is dependent upon it; and that should he return to his business, blind in one eye, he may soon, from a repetition of the casualty, become blind in both.

Passing over without notice, a number of injuries and diseases of the appendages of the eye, we shall dwell for a moment on inversion of the eye-lid.

"Entropium is a much more formidable affection than the preceding; (*ectropium*,) the constant friction of the inverted eyelid and cilia against the globe, quickly causing inflammation, and if not relieved, paniform opacity of the cornea, and total loss of vision. As a mere temporary condition, it occurs in some cases of opthalmia, the tumefied conjunctiva pressing out the orbital edge of the tarsus, while its ciliary margin is turned inwards by the action of the orbicularis, irritating the eye, and greatly aggravating the inflammation. When the disease is permanent, it sometimes arises from relaxation of the integuments, whether occurring spontaneously in advanced life, or from the injudicious employment of emollient applications. In other instances, it is seated in the tarsus, and appears as a sequel of inveterate psorophthalmia, or chronic catarrhal inflammation, ending in various morbid alterations in the texture or configuration of the palpebral margin. The upper and lower eyelid, are equally liable to be affected, and both may be inverted at the same time."

Dr. Littell suggests, that mild cases of this affection, may be treated with adhesive strips applied longitudinally, but this method is only paliative. He speaks with want of confidence, of the application of sulphuric acid, as proposed by Helling, and in our own hands it has not been successful. We consider it, indeed, as applicable to none but the slightest cases. Our author seems to think the horizontal excision of the loose skin of the eyelid, with the application of sutures, an operation that will generally succeed; but we believe with Mr. Guthrie, that in most cases it is necessary to cut through the ciliary cartilage and orbicularis muscle, near either cauthus, and also to remove an adequate portion of the loose skin between them. This operation is an improvement on that of Mr. Crampton; and as Mr. Guthrie's work is not generally in the hands of our western readers, many of whom are presented with severe cases of entropium, the consequence of endemic purulent ophthalmia, we shall venture to extract from Mr. Guthrie's work, his instructions for the performance of the operation. It is one which we have found it not difficult to perform, and it proves entirely successful.

"The operation I recommend, as equal to the cure of the worst cases, is to be performed in the following manner:—The head being properly supported, the eyelids are to be gently separated; the patient is to be desired to refrain from making any effort whatsoever, and the surgeon is to wait until he sees that the lids are perfectly quiescent. A small narrow knife, or one blade of a blunt-pointed scissors, is then to be introduced close to the external angle, and a perpendicular incision made, of from a quarter, to half an inch in extent, or of a sufficient length to render the eyelid quite free; the quiescent state of the lids, and especially of the orbicularis muscle, enabling the surgeon to cut closer to the angle than he otherwise could do, and thus to divide the ligament, or at least the extremity of the cartilage. Another incision is to be made, in a similar way, at the inner angle; this should not include the punctum lachrymale, but be external to it, as I have never found it necessary to divide it; for although the tears may continue to pass through the lateral canal into the sac, when the punctum has been included in the incision, they do not do so with equal freedom, and there is some observable deformity. The length to which the perpendicular incisions at both angles ought to extend, must now be decided upon by the appearance of the part; they must be continued, if necessary, by repeated touches with the scissors, until that part of the eyelid containing the tarsal cartilage is perfectly free, and is evidently not acted upon by the fibres of the orbicularis muscle, which lie upon it. This frequently causes the incisions, and especially the internal one, to be longer than is usually supposed to be necessary.—The part included in the incisions is now to be completely everted, and retained by the fore finger of the operator's left hand against the brow of the patient; when, if any lateral attachment be observed, acting upon, and drawing, or confining the lid, it is to be divided, which is in fact still elongating the incision. On letting the eyelid fall on the eye, the edge of the tarsus and the hairs will frequently appear in their natural situation, in consequence of the relaxation of the angles, which bound them down; but if the tarsal cartilage has become altered in its curvature, this will be immediately perceived; it will turn inwards at its ciliary edge, and be completely bent at its extremities, more especially at the inner one, where it is more powerfully acted upon by the musculus ciliaris. On desiring the patient to raise the lid, he readily attempts it, but the action of the levator, in such cases of vicious curvature, causes the cartilage partly to resume its situation; and, on examination, the curve will be observed to be so permanently vicious for about an eighth of an inch at each extremity, and especially at the inner, that it cannot be easily induced to resume its actual situation. When this is the case, the cartilage is to be divided, exactly at the place where it is bent, in its length, and in a direction at a right angle with the perpendicular incision. The portion thus slit is only connected with the common integuments of the eye-lid; and although this incision does not exceed one-eighth of an inch, at both extremities, and in general is only necessary at the inner, it enables the surgeon more readily to remove the altered curvature of the part. Mr. Crampton recommends that the two inner points of the perpendicular incisions should be now united by the subsection of Ætius and Paulus Ægineta, that is, by a division of the conjunctiva, which he supposes to be in a contracted state; but I have always found this division to be useless, and even injurious. It is useless, because it has no influence on the part, for a simple division of that kind unites or fills up in a very short time; and it is injurious, because, if made across the cartilage, it is apt to form a ridge of cicatrix which irritates the eye, and of which, among others, I have seen one particular and incurable instance. The operation being thus far accomplished, a fold of skin is to be cut away from that part of the eyelid included between the incisions; three or four ligatures are then to be introduced, and the divided parts, from which the fold has been removed, are to be neatly brought together by the ligatures, each of which ought to be twisted, and then fastened to the forehead by several short slips of sticking plaister, the ends being turned over the plaisters near the hair, and retained in that situation, to prevent their slipping. In raising the fold of skin, care should be taken to do it regularly with the fingers; it is also essential to the success of this part of the operation that it be done as close as possible to the margin of the eyelid. It may then be grasped by the forceps of Beer, which have transverse pieces, slightly curved for the purpose, at their extremities, and close with a spring. The piece thus included, which need not be large, may be cut away at one or more strokes of a large pair of curved or straight scissors. The ligatures should be inserted first at each angle; and when the vicious curvature is considerable, I not only pass it through the skin, but take care that *the internal* one shall include, at its lower part, the *outer edge* of the margin of the eyelid, which, from its firmness, retains that ligature much longer than those which are passed through the skin only, and tends to prevent the possibility of a relapse. The ligatures thus placed are to be equally drawn up on the forehead, until the eyelid is *completely everted*, when they are to be fastened as directed. In order to prevent any attempt at union but by granulation, or a filling up of the incision, the edges are to be slightly touched with the sulphas cupri; the eye and eyelids are now to be carefully cleansed; a piece of lint, spread with the unguentum cetacei, is to be placed upon them; a small compress is to be put under the edge of the eye-bone and orbit; a retaining bandage covers the whole, and completes the different steps of the operation. When the disease is not considered of an inveterate nature, the ligatures may be introduced through the fold of skin, without cutting any portion away. They give little additional pain, and are infinitely more effectual than any other suspensorium with which I am acquainted, but they ought to be assisted by strips of adhesive plaister placed between them.* The operation, accomplished with all the care I have described, will still fail, if equal attention be not daily paid to the subsequent dressing, on which, indeed, more depends than on the operation itself; so much, indeed, that I am disposed to consider inattention to it the most certain cause of failure. In more than one hundred operations that I have performed, I have never known inflammation or other bad consequences ensue. The patient is to be kept perfectly quiet until the next morning, when the bandage and lint are to be removed, the eye carefully fomented and cleansed with warm water, and the dressings replaced. On the second day, great attention must be paid, that the ligatures keep the lid sufficiently raised; and if any union has taken place by adhesion at the angles of the incisions, it must be broken through with the probe. On the third day, the plaisters, attaching the ligatures to the forehead, will in general require to be exchanged. The ligatures themselves must be supported by straps of plaister placed vertically between them; the edges of the incisions should again be touched with the sulphas cupri, or separated by the probe. The great art of the cure now consists in causing the incisions to be filled up by granulations only, so that the eyelid may be lengthened as much as possible, which can only be effected by a continuance of the means indicated. In a few days more, and especially by the continued elevation of the lid, the ligatures cut their way out, during which period the eyelid is gradually lowered, and by the time the incisions have filled up, it will have resumed its natural situation, and the cure will be completed. There will, in general, be an indentation in the ciliary margin of the tarsus, where the incisions were made, but this is sometimes scarcely, or not at all perceptible, and never detrimental. If the patient should have kept the lids closed at the commencement of the operation, by the action of the orbicularis, the first incision may not have been made as close as might be desired to the angle of the lids; and in this case a hair or two at that part may not afterwards obtain quite its, or their original direction, and cause some inconvenience; but this does not often happen, as these hairs are usually carried clear of the eye.

The operation on the under eyelid is analogons to that on the upper, but is less severe, as the parts are more simple. It consists, in inveterate cases, of a perpendicular incision made with the scissors at the outer angle, and carried downwards to such extent as will perfectly relieve the inversion. This incision, by dividing the fibres of the orbicularis at that part, as well as the conjunctiva, renders the muscular fibres powerless; and when it is properly made, the patient is incapable of moving the lid. One ligature is now to be inserted at the margin of the lid, and the threads fastened below the jaw by sticking plaister so as to keep the eyelid everted. The edges of the incision are to be touched with the sulphas cupri or the argentum nitratum to prevent union, and care must be taken daily that the part fills up by granulation.

* In order to ascertain how much skin ought to be cut away, the operator should first raise a fold with his fingers, and desire the patient to open the eye. If the fold be correctly made, the eyelid will be raised very readily to its proper distance; if the fold be too small, the eye will not be sufficiently exposed. In raising the fold of skin, more should be taken from the centre than from the extremities, so that, when it is removed, the wound may be of an oval shape.

If any union should have taken place from the ligature slipping, it must be broken down by the probe. In the very worst cases, the inner angle is to be divided and treated in the same manner, the integrity of the punctum lachrymale and lateral canal being observed. In more recent and less severe cases, the simple division of the outer angle will be sufficient, without any ligature, care being taken to prevent a too rapid union. I rarely recommend the use of the acids on the lower lid, as the operation is so very slight that the most timid need not fear it. Mr. Saunders notices an inversion of the lower lid, arising, as he supposes, from a thickened state of the conjunctiva, where it is reflected from the eyelid to the ball of the eye, and forming a roller at that part, over which, by the strong contraction of the orbicularis, the eyelid is turned and lodged between the projecting conjunctiva and the eye. If this be neglected, he says, it thickens and indurates to a very great degree; and he recommends its removal, and the subsequent application of a compress to carry the orbital edge of the tarsus inwards. I object to this operation as likely to give rise to a subsequent inversion, as well as to cure it, and have either not met with the case as described by Mr. Saunders, or have found the more simple operation I have described equally successful.

The subsequent treatment of the eye is much less than might be supposed, the principal cause of irritation being removed, *viz.* the inverted hairs, the inflammation gradually subsides, the conjunctiva assumes from day to day a more natural appearance, the opacities of the cornea diminish, and such alterations only, as are found to be permanent after the cure of chronic inflammation from other causes, remain as indelible marks of previous disease. Mild stimulants, such as the solutio opii Battlei; the liquor plumbi subacetatis; the vinum opii, made without spices; the tinctura ferri muriatis, diluted with two parts of distilled water; the solutio argentri nitratis, of from two to four grains, to an ounce of distilled water, are the best applications after the incisions are healed. Where the conjunctiva is thickened, or granulated, or fungous, the latter solution dropped into the eye twice a day is very useful; and in more inveterate cases, a saturated solution of the sulphas cupri in distilled water applied with a camel's hair brush, and well washed off with plain water, is often of essential service. In every case, some mild ointment should be applied to the edges of the lids at night, and any secretion, which may have arisen and become hard, should be removed by the aid of warm water in the morning. Indeed, the lids, whilst any vitiated secretion remains, should be carefully examined *every* morning, and great attention paid that any hair, which has changed its direction from having been clogged by the discharge, be replaced in its situation, or pulled out, if it appears to resume its place with difficulty. The best ointments are, the ung. cetacei, the ung. zinci oxydi, the ointment of Janin, the ung. hydr. albi gr. 2 ad ʒj, the ung. hydr. et oxydi rubri, six times reduced with the ung. cetacei, assisted during the day by the occasional use of any of the mild astringent lotions.

By the means, then, which have been thus fully described, I have reason to believe that a disease, which has been an opprobrium to surgery since the time of Hippocrates, may be always cured, under whatever name or description it may be included; and the six following cases will, I trust, be sufficient to remove any doubts, which may remain even in the minds of the most incredulous, whilst they will also justify the prolixity of detail which has preceded them."

Passing over a number of less formidable affections of the ophthalmic appendages, we come to diseases of the organ itself; and first, of those which chiefly affect the CONJUNCTIVA. This membrane seems to be sero-mucous. The portion which covers the cornea, partakes of the serous character, and has like the class of membranes to which it is allied, greater sensibility to external impressions, than the surrounding parts, which have greater affinity to the mucous membranes. That the organization and vital properties of the two divisions, are not identical, may be known from the fact, that inflammation often ceases, as it reaches the margin of the cornea.

Ophthalmia, in its common signification, is synonymous with conjunctivitis. We shall generally employ the latter term, as more definite in its meaning. There are several varieties or species of this affection, some of which result directly from applications to the membrane—others from disorder in some other part of the system. To the former head, belong all the cases which depend on injuries of the membrane from external violence, by mechanical and chemical agents; sudden vicissitudes of temperature; and the contact of gonorrhœal poison:—to the latter belong malarious, dyspeptic, strumous, rheumatic and several other distinct species of conjunctivitis. Thus the number of cases of inflammation in this membrane is very great, and the treatment which they require, exceedingly various. The general diagnosis is easy, but the particular diagnosis is often difficult. An intolerance of light is common to the whole, showing an increased sensibility in the retina; and, such is the relation between that tissue and the one we are now considering, that the action of an intense light, or a moderate light too long and steadily continued, as in artisans and students, will produce conjunctivitis. Dr. L., following in the footsteps of the systematists, recognises purulent conjunctivitis as a distinct variety; but a puriform secretion seems to us to mark an intense, rather than a distinct inflammation.

When the disease originates from external injury, the offending cause is often washed away by a flood of tears—the secretion of which is wisely promoted by the action of foreign bodies upon the conjunctiva. Of the manner of removing those which penetrate into the cornea we have already spoken.

Now and then, sand and dust and lime, are thrown within the eyelids, and if the physician be called at an early period, he should co-operate with the lachrymation, by introducing the point of a small syringe underneath the lids, and throwing in a liberal quantity of tepid water with mucilage or milk, and conclude his operation by injecting olive oil which is not rancid, sweet cream, or the oil of almonds. Sometimes a small stick, will become so disposed behind one or both of the lids, as to be invisible, unless they are completely everted. Our author has quoted a case, in which Dr. Monteath removed a piece of wood three quarters of an inch long, from behind the upper eyelid, where it had lodged for five months, without producing more than a slight inflammation. A similar case occurred in our own practice. A child had its eye injured by a blow from a chip. When we first saw it, the inflammation and swelling were too great to admit of an examination; and it was not until an active antiphlogistic treatment had been pursued for many days, that they were sufficiently reduced, to enable us to see a piece of wood, more than half an inch in length, lying vertically over the caruncula lacrymalis. On removing it with a pair of forceps, the inflammation immediately ceased. We have no doubt that the sight of many eyes has been lost, by foreign bodies of this kind remaining behind the lids; especially in children, who submit with great reluctance to the examinations which such cases require.

The treatment of conjunctivitis from external violence, is purely antiphlogistic. When, in plethoric habits, the inflammation runs high, copious venesection is indispensable: in milder cases, leeches or cups may suffice for this branch of the treatment. The incessant application of tepid water, the patient being laid on his back, with his head and shoulders raised, is far preferable to cataplasms of bread and milk, elm bark, or alum curd, so often made to compress and irritate the eyes. Saline cathartics are decidedly proper. Great exclusion of light, but not total darkness, is requisite. Under this treatment (abstinence from food and stimulating drinks being of course en joined), the inflammation will generally yield; but if it should not, recourse ought to be had to antimonials, of which tartar emetic is the best; and it should be administered in grain doses, so as to produce on the system a decided contra stimulant effect. Blisters, in due time, are beneficial; but we cannot commend their application over the eyelids, as recommended by some writers; for they compress the eye, and confine the tears and mucus behind the lids. A salivation is often resorted to in these cases; but we prefer tartar emetic to calomel, and our patients will generally concur in this preference. In the declining stages of this inflammation, Dover's powder, or some other combination of narcotic and nauseating medicine, will afford great comfort to the patient, and contribute, at the same time, to the removal of the inflammation.

In this stage of the disease it is, that stimulating and astringent collyria are proper. Employed prematurely, they are as pernicious as they are now beneficial: a fact that is sometimes not well considered in practice. Nor do we always consider, that when the time arrives for their use, they may be employed of great strength, and are of little value without. Many years ago we were instructed on this point by Sir Everard Home, in a paper published in the Transactions of the Royal Society, on the use of the gall of fishes and other varieties of acrid bile, in certain stages of opthalmia; a practice which we traced up to the ancient Hebrews. Our preceptor, in times past, was in the habit of triturating opium with acetate of lead in hot water, and filtrating a clear solution from the dregs and the copious precipitate. It *may* be, that the acetate was decomposed, and the water impregnated only with certain principles of the opium; but we think that this solution was decidedly more efficacious as a stimulating collyrium, than any aqueous solution of that drug alone, which we have seen tried. Of mineral solutions, that of the nitrate of silver has, in our hands, been most efficacious; and next to it we could place the solution of sulphate of copper, and bi-chloride of mercury. Whichever is used, should be strong enough to occasion smarting, and a copious secretion from the lachrymal glands and the conjunctiva. Thus the congestion is carried off, absorption promoted, and a new action introduced into the diseased parts.

Gonorrhœal conjunctivitis, is the effect of gonorrheal virus accidentally applied to the eye. Our author thinks also, of the leucorrhœal discharge; but when we consider how many females are affected with the latter disease, in proportion to the number who are seized with this variety of ophthalmia, we cannot but doubt the correctness of the opinion which he has adopted. The specific diagnosis of gonorrhœal conjunctivitis, is by no means clear. A limitation to one eye in the beginning, suddenness of attack, and excessive violence in the symptoms, are perhaps the characteristics most to be relied upon. If not speedily subdued, the vision is evidently lost.

"General and local depletion to an extent commensurate with the urgency of the symptoms, active purgatives, free scarifications of the tumefied conjunctiva, and other auxiliary remedies, are proper in every instance; but the nitrate of silver is our principal reliance, and should be applied in substance to the chemotic swelling, and to the inner surface of the palpebræ. Even Mr. Lawrence, though he recommends the freest adoption of antiphlogistic measures, is constrained by the repeated instances of their failure, and the melancholy ravages of the disease, to assent to the employment of this remedy. Mr. Middlemore objects to the use of strong local stimuli in the commencement, but when the acute symptoms have been decidedly dimished by bleeding and other means, the discharge lessened, and the florid vascularity of the conjunctiva is superseded by a pale flabby appearance of that membrane, he recommends the nitrate, either in the form of ointment or strong solution. In many cases, however, such delay would be ruinous; and after one large bleeding, cupping from the back of the neck, free scarifications, and the operation of a brisk cathartic,—remedies which may be employed in rapid succession—recourse should be at once had to the caustic.

"Baron Dupuytren strongly advises the insufflation of calomel once or twice a day, and the liquid laudanum of Sydenham dropped into the eye at night."

It is remarkable that no mention is made of either tartar emetic or calomel in the treatment of this affection. After due sanguineous depletion, they should be administered in combination, one grain of the former and ten of the latter, every two or four hours, until a salivation is induced.

Under the head, purulent conjunctivitis, Dr. L. treats of the epidemic and contagious varieties. There is much obscurity around their ætiology. The epidemic ophthalmia of Egypt and other warm climates, especially affecting armies, has been ascribed to the intense action of the reflected solar rays, but this is doubtful. In this country, a disease of nearly the same symptoms arises, and, occasionally, affects great numbers, under circumstances exactly opposite; for it is epidemic in shaded and humid situations, in autumn. In short, it appears under the topographical conditions which favor the production of bilious fever; and, like the latter, becomes less prevalent with the clearing and cultivation of the country, although the brightness of the sun's light is thereby increased.

Dr. L. seems to think, purulent conjunctivitis contagious. This opinion is by no means uncommon in the West. Many people even believe, that it is caught by looking at the eyes of one affected! We have not had many opportunities of personal observation on the alledged contagiousness of our endemic ophthalmia; but recollect to have had a patient, with opacity of the cornea, who was one of nearly thirty laborers on the Miami canal, who were at one time affected with this malady. It might, however, have been gonorrhœal; as the life and habits of such operatives are favorable to the introduction and spread of that variety: the whole of them, moreover, were alike exposed to a malarious atmosphere. We have often known it seize upon all the members, particularly the children, of a family, in the *new* settlements of the West; but these, again, were subjected to the same atmospheric influences.

When the autumnal conjunctivitis of the West is combatted by mere topical remedies, it generally terminates in a disorganization of the anterior part of one or both eyes; and the number of cases of blindness, or impaired vision, from this cause, in the valley of the Mississippi, out of the principal towns, in which it seldom prevails, is very great. The treatment should be substantially the same that has been laid down for gonorrhœal ophthalmia. But its apparently malarious origin, should be kept in mind. The sufferings of the patient are almost always greater at night than in the day, and in this respect it is analogous to our remittent bilious fevers. It frequently refuses to yield to mere antiphlogistics; but disappears under the use of the bark, or the sulphate of quinine.

Infantile conjunctivitis, with purulent secretion, is by no means an uncommon disease in the West. Our author regards it as the offspring of the contact of an acrid vaginal secretion, or of the inordinate action of light. But how can it be known that the former has been applied? And we have seen nothing to justify the latter conclusion. Whatever may be the cause, it is, in reference to the vision of the child, a most dangerous affection; sometimes producing a turbid state of the aqueous humor, at other times an aperture in the cornea, from ulceration; more commonly an opacity of that membrane; and now and then, a staphylomatous protrusion of the front of the eye. We have not often seen any high degree of constitutional disorder awakened, and irretrievable blindness may be produced, when no danger is suspected. The swelling of the lids is generally so great, that it is difficult, either to inspect the conjunctiva, or to introduce a collyrium upon it. Moreover, when introduced, the quantity of purulent matter is often so great, as seriously to interfere with its action upon the inflamed membrane. Hence the syringe is an important instrument in the treatment of this malady. Its point should be long and smooth, and introduced near the outer angle of the eye, it should be carried forward behind each lid, pressing gently against them so as to avoid the eye-ball. In this way the morbid secretions should be frequently washed out, and, immediately afterwards, a solution of nitrate of silver in rain, distilled, or rose water, thrown in. This may, generally, be of the strength of four grains to the ounce—weaker when the child is of a full and phlogistic habit, stronger when of an opposite diathesis. When it runs into either of these extremes, appropriate constitutional treatment is required; but, in general, our reliance has been on topical applications.

In all the varieties of ophthalmia of which we have spoken, when an ulcer is established on the cornea, we know of nothing, which can arrest it, but the solid nitrate of silver, as recommended by Mr. Saunders. Its action should be limited to the affected part, which can be done by dashing water into the eye as soon as the dissolved caustic begins to spread over the ball.

For the thickened and carunculous state of the palpebral conjunctiva, which so often follows acute, or is connected with the chronic inflammation of that membrane, the best remedy is the crystalized sulphate of copper, drawn frequently over the affected part.

Strumous conjunctivitis should be contemplated under two aspects;—first, the inflammation immediately excited by cold or some external violence, in an individual having a scrofulous habit: second, it may arise spontaneously, in such habits. In the latter case, it is sometimes preceded or followed, by other local affections. This affection is not to be combatted by antiphlogistics, especially when it arises independently of external agency. Dr. L. justly remarks, that the constitutional treatment only, can be relied upon. Appropriate means of correcting a morbid functional condition of the chylopœetic organs, must, from time to time, be employed.

"A light nutritious diet, regular exercise in the open air, the tepid salt or shower bath, frictions over the body with the flesh brush or a coarse towel, diaphoretics, and whatever has a tendency to promote the cutaneous functions and invigorate the system, will also exert a beneficial influence. The sulphate of quinine is an important remedy, and often displays a striking power over the disease, allaying the morbid sensibility, and removing the inflammatory symptoms in a very remarkable manner. It may be exhibited in any convenient form, and in quantity varying according to the age of the patient, and the urgency of the symptoms. To a child one or two years old, half a grain to a grain may be given three or four times a day. In conjunction with the sulphate of iron, the syrup of orange peel, and a few drops of sulphuric acid, it forms an elegant prescription. For persons of more advanced age, or where a treatment more decidedly tonic is demanded, it may be also advantageously combined with columbo, and the precipitated carbonate, or the proto-sulphuret of iron; and it is in this combination, that it will generally be found most serviceable. Iodine, the diluted sulphuric acid, and the liquor potassæ, have also been recommended; the article first mentioned, is occasionally employed with good effect when there are scrofulous tumours in other parts of the body, and the two last may sometimes be usefully prescribed in cases attended with anorexia, and an irritable condition of the system, but as a general remedy they are all greatly inferior to the sulphate of quinine."

Our own experience leads us to the same conclusion, as to the powers of iodine. The best preparation, is the liquor ferri hydriodatis, which unites the tonic qualities of iron, to the specific alterant powers of iodine.

Great, as is the intolerance of light in this malady, the patient should not be indulged in the luxury of a dark room; for the susceptibility is thereby augmented, and the powers of his constitution still further impaired. Active exercise in the open air, is one of the most important remedies within our reach.

Our author has a few pages on the ophthalmias connected with small pox, measles, and scarlatina. Our own experience teaches us, that they should all be managed in the same way, and that the treatment should be, chiefly, preventive. A dark room, and the incessant application of cold water to the eyes, are the simple means, that will seldom disappoint our expectations.

Physicians are frequently consulted for conjunctival inflammation, more or less acute, in persons of high health and plethoric habits, when no obvious exciting cause has existed. A reduced and vegetable diet is indispensable in such cases; most of which will yield to it alone, and to it only.

Other cases, of constitutional origin, are dyspeptic. For the most part, there is an absence of the symptoms of plethora and inflammatory orgasm. These require antacids, gentle chologogues, and a temperate diet.

Once more. Intemperate drinking, as all the world has seen, displays itself in an obstinate, though mild conjunctivitis, remediable only by abstinence from the remote cause.

We have dwelt for some time upon affections of the conjunctiva, with the less scruple, because they are exceedingly frequent in the West; and generally fall into the hands of those who are not professed ophthalmic surgeons. Every physician should qualify himself, for treating them according to the most approved principles.

Next in order come diseases of the cornea. Corneitis, or inflammation of the transparent tunic of the eye, occasionally exists with but little disease in the conjunctiva. It is sometimes strumous, and discloses itself by white nebulæ, which shift their position. At other times it is variolous. More commonly it results from mechanical injury. The treatment of corneitis is substantially the same with conjunctivitis.

Opacities of the cornea, the consequence of these and other inflammations of that membrane, are always slow in disappearing, and, often, so organized, as to be immoveable. Their cure is sometimes attempted while a considerable degree of inflammation remains, which is improper.

"Every source of irritation having been carefully investigated, and as far as practicable removed, a variety of stimulating applications are recommended with the view of promoting the absorption of the effused lymph; such as the solution of the nitrate of silver, the sulphate of copper, or zinc, the bi-chloride of mercury in the proportion of two grains to the ounce, and the vinum opii, pure or diluted. These solutions, gradually increased in strength according to the irritability of the eye, should be perseveringly employed; and as their effect is diminished by repetition, it will also be proper to vary them occasionally. In recent cases, the ung. hydrargyri nitratis, applied to the seat of the opacity by means of a camel's hair pencil, is an excellent remedy. Leucoma, and the denser forms of albugo, though they cannot be entirely removed, may often be much diminished by the absorption of the lighter deposition around their circumference. For this purpose, the ointment of the nitrate of silver, repeated at intervals of two or three days, in conjunction with the internal administration of mercury, counter irritation, &c. may frequently be employed with decided advantage. The ointment of the hydriodate of potash is also useful under these circumstances. BARON DUPUYTREN relied chiefly on the insufflation of the red precipitate, according to the following formula:

℞ Hydrarg. oxid sub. gr. x.

Zinci oxid. impur. gr. xx.

Sacch. alb. pulv. ʒij. Misce.

or the oxide of zinc and sub-muriate of mercury,

℞ Zinci oxid.
Hydrargy. sub mur.
Sacch. alb. pulv. a a part. 2 qual. Misce.

The application is made twice in the twenty-four hours, and according to that eminent practitioner, recent opacities are thus removed in two or three weeks, while those which are more deeply seated and extensive, involving nearly the whole cornea, and entirely preventing the transmission of light, may be dispersed in as many months.

When the effused lymph has become organized, and has acquired a dense, shining appearance, surgical treatment is of no avail, and it is unnecessary to annoy the patient by persisting in the employment of remedies which are incompetent to the removal of the disease. In such cases, if the opacity be partial, and situated near the centre of the cornea, vision may be much improved by the daily employment of belladonna."

In the opacities following on badly treated miasmatic ophthalmias, we have seen a very sudden absorption promoted, by a gentle salivation; and, occasionally, the administration of the bark, is attended with beneficial effects.

Stuphyloma, or a protrusion forward of the opake cornea, is another sequela of ophthalmia; and is exceedingly common in the western states, as a consequence of endemic autumnal sore eyes. The cornea loses its regular figure, is thickened, and at last projects beyond the eye-lids. The quantity of aqueous humor appears to be greatly augmented. By irritating the eye-lids, a conjunctival inflammation is kept up.—Complete excision of the cornea, has been practised by some operators, while others have preferred a simple puncture with a cataract needle. The former is more extensive than most cases require, and may be followed by an escape of the lens and even the vitreous humour; while the latter often closes prematurely; we have, therefore, preferred the removal of an oval portion of the most projecting part of the cornea, which may be conveniently done with a hook and scissors. The fluids escape as fast as they are secreted, the cornea, iris and capsule come together and adhere, and a tuber, in all respects adapted to the reception of an artificial eye, is thus produced. Many of our brethren in the country, decline the performance of this operation, which is, in fact, one of the most simple to which they could be called.

The sclerotica is much less liable to disease than the conjunctiva, or even the cornea. It requires an experienced eye to distinguish by the appearance, between conjunctivitis and sclerotitis.

"Inflammation of the sclerotica is characterized by the peculiar appearance and distribution of its minute vessels, which exhibit a pale red, or pinkish colour, and run in a radiated direction to the margin of the cornea, around which they form a wreath or plexus, more or less distinct—in some cases slightly encroaching upon its surface. The pupil is contracted, and less active than usual, indicating the presence of iritis; and when this complication exists in any considerable degree, the zonular arrangement is more strongly marked. The pain is dull, aching and tensive, often less severe in the globe than in the surrounding parts, and is greatly aggravated at night, the patient being comparatively free from uneasiness during the day; headache and hemicrania are also occasional symptoms. The vascularity of the conjunctiva is, in general, inconsiderable, a few dilated vessels only being perceptible; and there is usually little intolerance, or lachrymation, but in severe cases, these symptoms are present in a high degree, accompanied with fever, and other manifestations of constitutional suffering. The inflammation is often limited to one eye, and frequently alternates with rheumatic affections in other parts of the body, or makes its attack as they subside. It is tedious in its progress, less decidedly influenced by remedies than disease of the other tissues, and leaves an irritable condition of the organ which renders it liable to relapse from slight causes.

Sclerotitis is most common about the middle period of life, and is generally caused by exposure to cold. Allusion has been made to its connection with inflammation of the fibrous textures, and it may now be stated that it is also occasionally seen in conjunction with gonorrhœa. Iritis, glaucoma, attenuation and staphyloma of the sclerotica, are among its morbid terminations."

The cases of sclerotitis which have fallen under our observation have generally been rheumatic. The reader will recollect the fibrous structure of that membrane. Local applications are of no great efficacy in this disease, the treatment of which should be substantially the same as that of rheumatism.

Dr. L. has a page on choroiditis, which, being far from a common affection, we shall pass over, to copy his summary of the treatment requisite in acute retinitis; the most painful, and in reference to vision, one of the most dangerous of all the ophthalmic inflammations.

"Though extremely dangerous, retinitis is not always destructive to vision, if the appropriate remedies are vigorously employed before the sight is wholly extinguished. The most active antiphlogistic treatment is demanded, and no time should be lost in carrying it to its fullest extent. The patient should be bled ad deliquium, and the vein must be re-opened if a decided impression have not been produced by the first operation; cupping from the temples and back of the neck, active cathartics, &c., should be prescribed in quick succession.—Mercury is an invaluable remedy in this form of ophthalmia, and should be exhibited in such manner as to produce its constitutional impression with the least possible delay. Counter irritation will also be useful, the light must be carefully excluded, and every part of the antiphlogistic system rigidly enforced. In the management of this formidable affection, the surgeon should bear in mind the suddenness of its onset, and the rapidity of its progress; irreparable mischief is sometimes produced in a very few hours; and although the extent of the depletion must be regulated by the constitution and state of the patient, it ought in every instance, to be carried as far as can be done consistently with a due regard to these circumstances."

Chronic retinitis is so often the cause of amaurosis, that our author treats of them in the same section. His summary of the symptoms is worthy of being transcribed entire:

"Acute retinitis, whether owing to the vehemence of the attack, neglect, or insufficient treatment, is extremely liable to terminate in loss of sight; but there is a chronic inflammation more frequent in its occurrence, which may be equally destructive to vision, and as it is perhaps the most common cause of amaurosis, may be properly included in the description of that disease.

When amaurosis occurs as a consequence of chronic inflammation of the retina, the usual indications of vascular excitement are present, though in a much slighter degree than in the acute form; the patient complains of pain or uneasiness in the eye, accompanied with a sense of heat, dryness, and a morbid sensibility to the impression of light, which, however, may not amount to actual intolerance. He evinces an aversion to use the organ, and is often annoyed by muscæ volitantes and ocular spectra of different kinds. The sensibility of the membrane is gradually lessened by interstitial deposition, and vision is impaired in every intermediate grade, from slight dimness to total loss of sight; objects at first appear obscure, as if enveloped in mist or smoke, are confusedly blended together, and in some instances are only partially discerned; while in others their shape is variously distorted, or there is an erroneous perception of colour. Strabismus and double vision are frequent symptoms, and it may happen that where the central portion of the retina is quite insensible, the patient is still capable of seeing objects situated latterally with tolerable distinctness. The ocular spectra assume various forms; exhibiting an appearance as of net-work or gauze, dark motes, or pellucid serpentine tubules or striæ, and not unfrequently flashes of light, sparks, and other luminous bodies. They may be either fugitive and occasional, or fixed and permanent; and are an unfavorable symptom, inasmuch as they usually indicate alteration in the texture of the retina. The pain, likewise, differs in degree, nature and situation; it is often accompanied with giddiness, and is frequently a prominent subject of complaint, though in many cases it is rather an uneasy sensation of fullness and distension, than a feeling of positive suffering. It may be either constant or otherwise, assumes sometimes the form of general headache or hemicrania, and is frequently confined to the brow and neighbouring parts. The pupil is usually dilated and immoveable, or if the iris still preserve any degree of activity, its motions are sluggish and limited in extent; instances, however, of regular contraction and dilatation are not very uncommon. If one eye only be amaurotic, the pupil may often be observed to dilate when the other is closed, and vice versa; it is very frequently irregular, and instead of its natural pure black colour, exhibits a dull, turbid, or horny appearance. When the amaurosis arises from general debility, or is symptomatic of irritation in other parts of the body, the pain and other indications of increased action, are much less apparent, and in many instances, there is, from the beginning, a diminished sensibility to light, the patient requiring a brilliant illumination of objects for the purposes of vision. Imaginary sensations about the eyes are not unusual, and the disease is sometimes complicated with paralysis of the levator palpebræ. In every instance where the amaurosis is complete and inveterate, the countenance loses its expression, and acquires a vacant unmeaning stare, which at once betrays the nature of the malady. Under such circumstances, also, the globe frequently has a tremulous, vacillating, or rolling motion.

The progress of amaurosis is extremely variable; it may be produced suddenly, or as is more frequently the case, may require months or even years for its full development. It is most common in the middle period of life, usually commences in one eye, and does not attack the other, until vision in that first affected, is either much impaired or totally destroyed; and may be either complete or partial, permanent, temporary, or periodical."

Without following him through the details of the treatment of this affection, which must necessarily be extremely diversified, and are always uncertain in result, we shall transcribe a paragraph on one of the remedies, as being new to many of our readers:

"Strychnia promises to prove an useful acquisition to our therapeutical agents in some forms of amaurosis. It is employed in the endermic method; the cuticle having been removed from a space above the brow by the previous application of a blister, one-fourth of a grain is sprinkled on the abraded surface. The quantity is gradually increased to two grains, and if it occasion much pain or irritation, it may be mixed with a little pulverized opium, or any simple substance. MIDDLEMORE and others speak of this article in terms of high commendation, but the effects which occasionally follow its employment, depend probably less upon its general impression, than its action as a local irritant."

The iris is frequently inflamed. It may become involved in the inflammation of other tissues of the eye, but an idiopathic iritis is not uncommon. Like those of the other serous membranes, its inflammations throw out a coagulating lymph, which sometimes plugs up the pupil, and often loads the membrane so as to diminish its mobility. The pupil generally becomes permanently contracted. As a consequence of these various lesions, blindness is often the ultimate result of iritis, whether acute or chronic. The treatment of the former is well summed up by our author in the following page:

"The principal danger in iritis, arises from the effusion of coagulable lymph—an occurrence which can only be prevented by the adoption of prompt and judicious measures. Venesection should never be omitted when the state of the constitution will permit its employment, and the repeated abstraction of blood by cupping, from the temples and back of the neck, with an active cathartic in the beginning, and the rigid observance of the antiphlogistic system, will always be proper. It is in this disease that mercury most conspicuously displays its remedial powers, in preventing the deposition, and promoting the absorption of lymph; it should be early administered in such quantity as will place the aystem fully under its influence with the least possible delay, and it is sometimes necessary to continue its impression during a period of several weeks. Salivation is not absolutely required in every instance, but it generally produces a more decided effect upon the symptoms, and is therefore advisable whenever they assume a threatening aspect. Frictions over the brow and temples with equal parts of the tinctures of belladonna and cantharides, or the liniment hydrarg. comp.* blended with opium, are often useful in allaying the severity of the pain; anodyne fomentations† may also be prescribed

---

* ℞. Ung. Hydrarg. Fort.

Adipis, a a, . . . . . . . . . . . ℥j.

Camphoræ,

Alcohol, a a, . . . . . . . . . . ʒij.

Aquæ Ammon. . . . . . . - . . . ℥i.

To the camphor dissolved in the spirits of wine, add the aqua ammoniæ, and the ointment previously blended with the lard.

† ℞. Capsul. Papav. Alb. . . . . . . ℥j.

Chamæmel. . . . . . . . . . . ℥ij.

Semin. Lini. . . . . . . . . . ℥fs.

Aquæ, . . . . . . . . . . . oiij.

℞. Fol. Stramon. Recent. . . . . . ℥iij.

Aquæ, . . . . . . . . . . . oiij.

Evaporate, in each of the above prescriptions, one third of the fluid, and strain.

with the same view, and particular attention should be directed to obviate the contraction of the pupil by means of belladonna, though it unfortunately happens that this narcotic exerts comparatively little influence over the membrane in its inflamed state. Counter irritation is inadmissible during the existence of the acute symptoms, but is an useful auxiliary after the active stage has subsided. MR. CARMICHAEL warmly recommends the oil of turpentine* when from any cause a substitute for mercury is desired, and its utility in such cases has been confirmed by the testimony of others. Where the iritis has assumed a chronic form, and the patient has been much debilitated by the previous treatment. the sulphate of quinine is sometimes eminently serviceable; and if under these circumstances, there should be a congested condition of the external vessels, some gentle astringent, or stimulating collyrium may be employed with advantage."

The treatment of subacute or chronic iritis is by the same means as acute, applied (except the counter irritation) with less energy. This variety is often associated with certain constitutional lesions—the syphilitic, rheumatic, and strumous—when the remedies adapted to the cure of those morbid states of the constitution, are indispensable.

From the beginning to the end of a case of iritis, it is important to keep the pupil dilated. An ingenious graduate of the University of Pennsylvania, Dr. Samuel Cooper, was the first to point out the power of narcotics in dilating the pupil. His experiments were made with stramonium, in 1797. Our author has introduced Dr. Walker's explanation of the *modus operandi*, which we shall transcribe:

"An ingenious explanation of the modus operandi of these substances is given by MR. WALKER. He supposes that the branches of the third and fifth pair of nerves, which unite to form the lenticular ganglion, preside, respectively, over the dilatation and contraction of the iris; the former being appropriated to the radiated, and the latter to the circular or orbicular fibres. Filaments from the same division of the fifth, are also distributed to the palpepræ and forehead, and through this nervous communication the narcotic influence is propagated to the sphincter fibres of the iris, which, by their extreme delicacy of structure, and the absence of any fixed attachment, are rendered liable to paralysis from a degree of narcotism which would not otherwise be perceptible. The contraction of the orbicular fibres

---

* ℞. Ol. Terebinth. Rect. . . . . . . ℥j.

Vitel. Unius Ovi. . . . . . . .

Terre simul, et adde gradatim.

Emuls, Amygdal. . . . . . . . ℥iv.

Syr. Cor. Aurant, . . . . . . ℥ii.

Tinct. Lavend. Comp. . . . . ʒiv.

Ol. Cinnam. . . . . . . . gtt. iv.

Dose, ℥i, three times a day.

being thus temporarily suspended, while those supplied by the third pair preserve their vitality unimpaired, the antagonism is destroyed, and dilatation is of course the result."

Dr. L. has devoted only seven pages of his manual to the methods for the formation of an artificial pupil; but in that brief space, has given a clear and intelligible account of the different operations for that purpose. They are referred to three different heads: first, *Corotomia*, or simple incision, with the knife of Cheselden, which has been improved in Wills' Hospital, to which he belongs, by giving it the shape of a sharp-pointed bistoury; or by the scissors of Maunoir: second, *Corectomia*, or the excision of a part of the iris, by dragging it with a hook or forceps, through a puncture in the cornea, or by cutting out a triangular section, the base of which is towards the ciliary ligament, with a pair of scissors: third, *Corodialysis*, or the separation of the iris from its sclerotic attachment with a curved cataract needle passed across the disk of the eye. We regard these operations as the most difficult which the opthalmic surgeon is called on to perform. The selection among them, must be made from considerations connected with the state of the eye, in each individual case; and the judgment of the operator, will appear in the manner in which his decision is made.

Diseases of the lens and its capsule, are next presented. Capsulitis, or inflammation of the capsule, is disposed of in a couple of pages; quite enough, we think, for a subject so obscure; though a thoroughbred German *ophthalmologist*, would expand it into a long chapter, the result of which would be, that nothing can be done, but to remove, by an operation, the cataract, which is the inevitable consequence of such a morbid action. To this operation, eighteen or twenty pages are devoted. They present a distinct though brief view of the varieties of cataract, and the different methods of operating; but our limits forbid our entering, at this time, on a subject of so much magnitude, and we shall ere long make it the topic of an entire article. The same want of space, must preclude us from noticing in detail, the remaining heads, of which the principal are, hydrophthalmia, glaucoma, synchisis oculi, ophthalmitis, or simultaneous inflammation of several tissues, atrophy, cancer, fungus hæmatodes, extirpation of the eye, strabismus, and the different *opias*.

These and other heads, are expanded according to their relative importance; and complete the Manual, in a way, which, in our humble opinion, constitutes it the best elementary and text book for students, before and during their attendance upon lectures, that has yet fallen into our hands. The style is simple and perspicuous; extraneous matters are excluded; the definitions are generally well expressed; and the diagnosis of almost every affection is adequately made out.

As our article is designed to encourage students and young physicians, in the study of ophthalmic diseases, we shall be excused for reminding them, that this department of the profession has been most discreditably neglected by us. Nothing is more common, than to hear physicians, and in this country they all profess to practise surgery, acknowledge their ignorance of diseases of the eye. But why should this be the case? We sincerely hope it will not be so always. The great importance of the organ, taken in connexion with the frequency of its maladies in the West, and the remoteness of many patients from those who have been careful to prepare themselves for this kind of practice, should inspire our students with a more conscientious ambition in this matter. Our medical schools, also, should awaken to *their* duty on this subject. We believe it to be a fact, that but few candidates for graduation are examined on diseases of the eye; and that many of them go forth with diplomas, unacquainted even with the diagnosis of its most common maladies. Not a little of this, it must be confessed, results, *ex necessitate*, from the short time allotted, both by teachers and pupils, to the study of medicine. If departments of medical knowledge *must* be passed over superficially, it is natural, and indeed proper, to neglect those, which, in reference to the preservation of life, are least important; but the loss of sight is a calamity of so much magnitude, that ignorance, from the hurry of preparation, should not excuse him by whom it is perpetrated.

In conclusion, we ought to state, that Dr. Little has appended to his work a comprehensive and valuable *formula medicamentorum*, selected from the best practical writers, which of itself would be worth more to the insulated practitioner, than the cost of the book. D.

